# Supplementary material for: A Vector-Based Computational Model of Multimodal Insect Learning Walks
Source: Biomimetics (Basel). 2025 Nov 3;10(11):736. doi: 10.3390/biomimetics10110736 (PMC12650027; doi:10.3390/biomimetics10110736)
Supplement: Supplementary file 1 [file biomimetics-10-00736-s001.zip › biomimetics-3867713-supplementary/FigureS1-S3.pdf]

Supplementary Figure S1

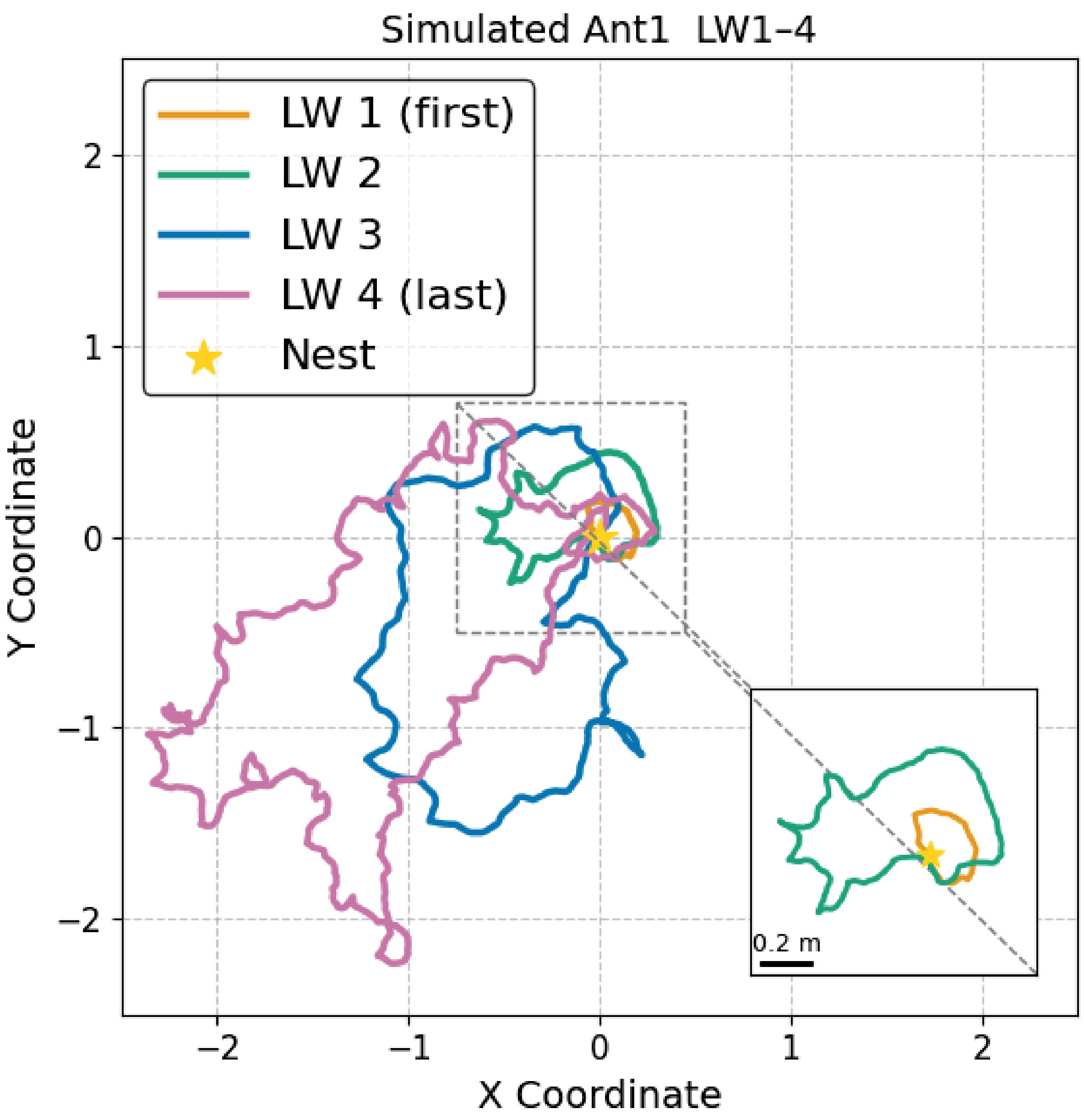

Trajectory evolution across four Learning Walks for a representative agent (Ant1). Inset shows zoom-in around the nest.

Supplementary Figure S2

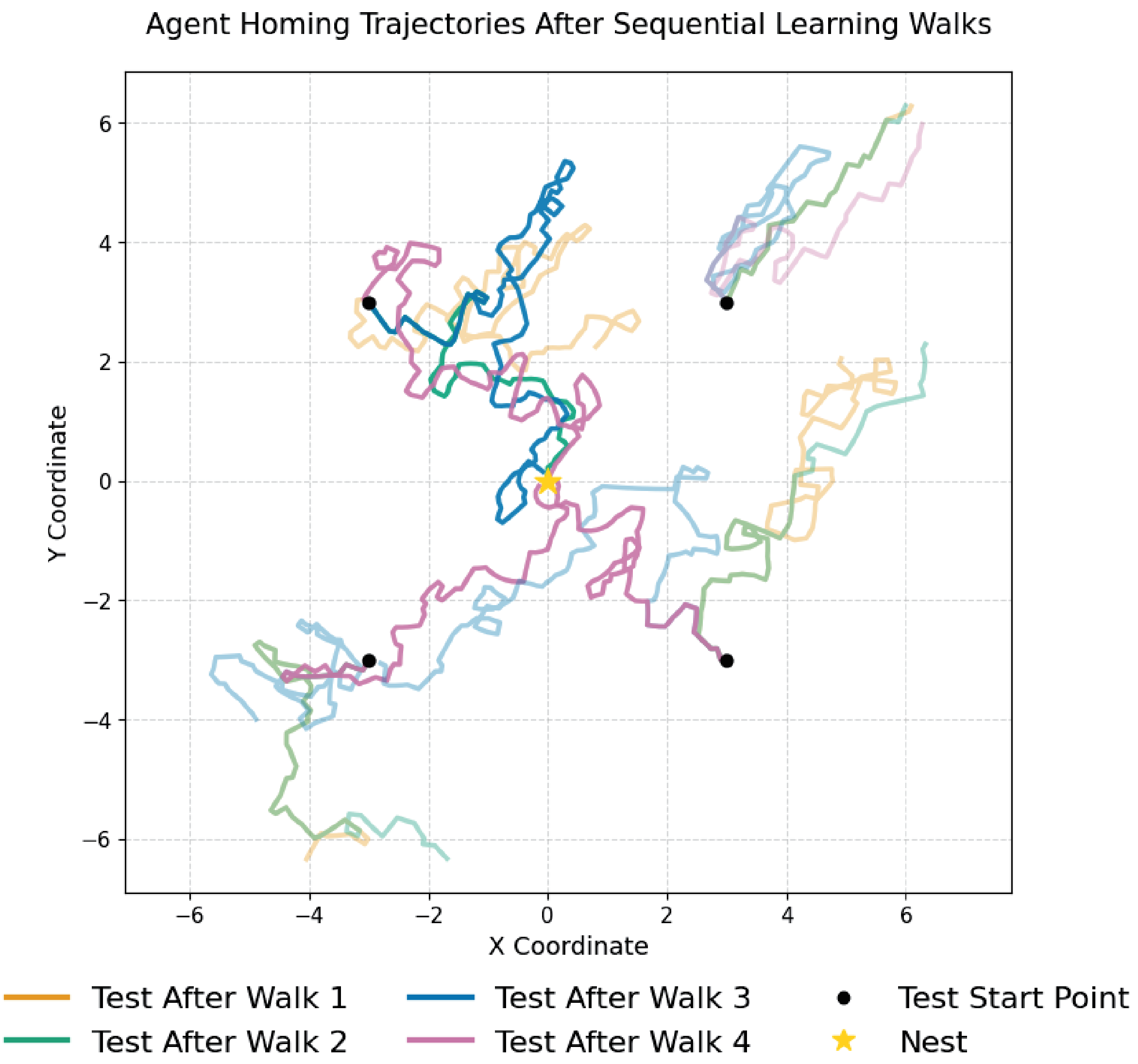

Homing trajectories of the agent after each sequential Learning Walk. Different colours represent the homing attempts following Walks 1-4.

Supplementary Figure S3

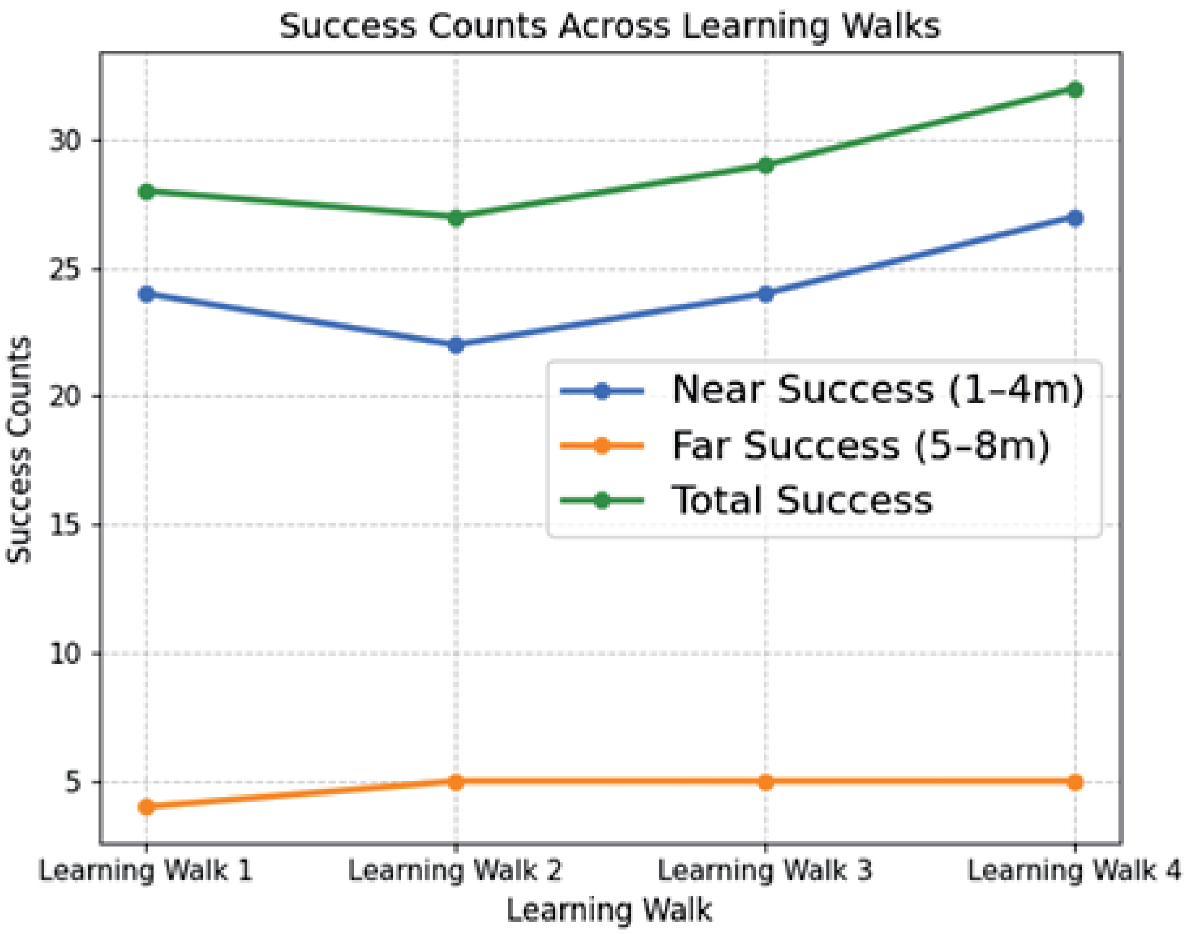

Success count statistics over Learning Walks, divided by near-range and far-range test distances.
