# Supplementary material for: A Vector-Based Computational Model of Multimodal Insect Learning Walks
Source: Biomimetics (Basel). 2025 Nov 3;10(11):736. doi: 10.3390/biomimetics10110736 (PMC12650027; doi:10.3390/biomimetics10110736)
Supplement: Supplementary file 1 [file biomimetics-10-00736-s001.zip › biomimetics-3867713-supplementary/TextS1.pdf]

1 Computation of Zernike Moments (ZM)

Zernike Moments (ZM) are image descriptors based on Zernike polynomials, widely used for image compression and familiarity estimation in navigation models. In this study, ZM is used to represent panoramic views in order to quantify visual familiarity. The computation steps are as follows:

1. **Image preprocessing:** We start from an input image Img_n, which is converted to grayscale, normalized, and mapped onto the unit disk with coordinates

$$r = \sqrt{x^2 + y^2}, \quad \theta = \arctan 2(y, x).$$

2. **Construct Zernike basis functions:**

$$V_n^m(r, \theta) = R_n^m(r)\, e^{im\theta},$$

where $n \in \mathbb{N}^+$ is the order, and $m \in \mathbb{N}$ is the repetition satisfying the conditions $|m| \leq n$ and $(n - |m|)$ even, to ensure the rotational invariant property.

3. **Radial polynomial:** The radial polynomial $R_n^m(r)$ is given by

$$R_n^m(r) = \sum_{s=0}^{(n-|m|)/2} (-1)^s \frac{(n-s)!}{s!\left(\frac{n+|m|}{2} - s\right)!\left(\frac{n-|m|}{2} - s\right)!}\, r^{\,n-2s}.$$

4. **Compute Zernike moments (continuous form):**

$$Z_n^m = \frac{n+1}{\pi} \iint_{x^2+y^2 \leq 1} f(x, y)\, V_n^{m*}(r, \theta)\, dxdy,$$

where $f(x, y)$ is the image intensity function and V_n^{m*} is the complex conjugate of the basis function.

5. **Discrete form for digital images:** For a digital image, the integral can be approximated by a double summation:

$$Z_n^m = \frac{n+1}{\pi} \sum_x \sum_y f(x, y)\, V_n^{m*}(\rho, \theta), \quad x^2 + y^2 \leq 1.$$

6. **Feature vector representation:** Select a set of low-order moments Z_n^m, and use their magnitudes $|Z_n^m|$ as a compact feature vector to form the "ZM memory." In the main text, A_i refers to these magnitudes $|Z_n^m|$, where the number of indices i depends on how many (n, m) pairs satisfy the constraints described in step 2.
