# Supplementary figures and images for: A Vector-Based Computational Model of Multimodal Insect Learning Walks
Source: Biomimetics (Basel). 2025 Nov 3;10(11):736. doi: 10.3390/biomimetics10110736 (PMC12650027; doi:10.3390/biomimetics10110736)

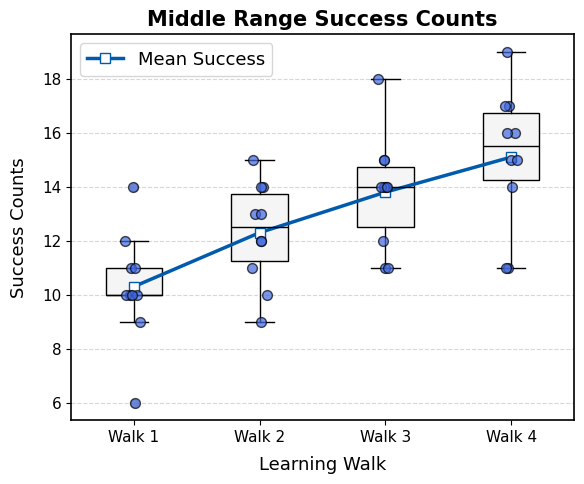

Supplement: Supplementary file 1 [file biomimetics-10-00736-s001.zip › biomimetics-3867713-supplementary/Supplementary_Statistical_Results/FigureS32.png]

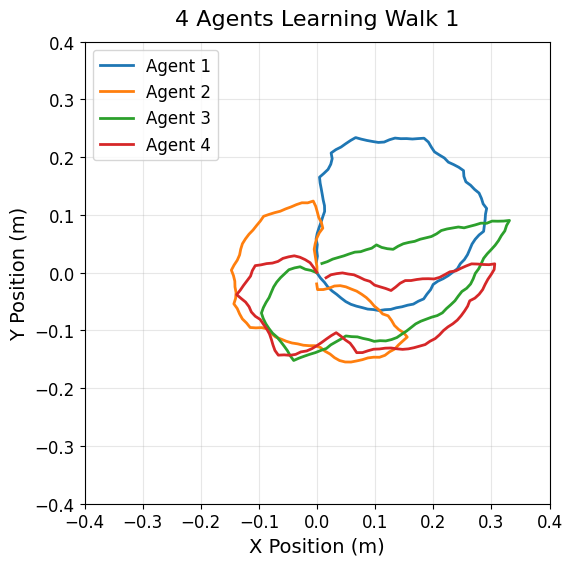

Supplement: Supplementary file 1 [file biomimetics-10-00736-s001.zip › biomimetics-3867713-supplementary/Supplement_Asymmetric olfactory field/FigureS4.png]

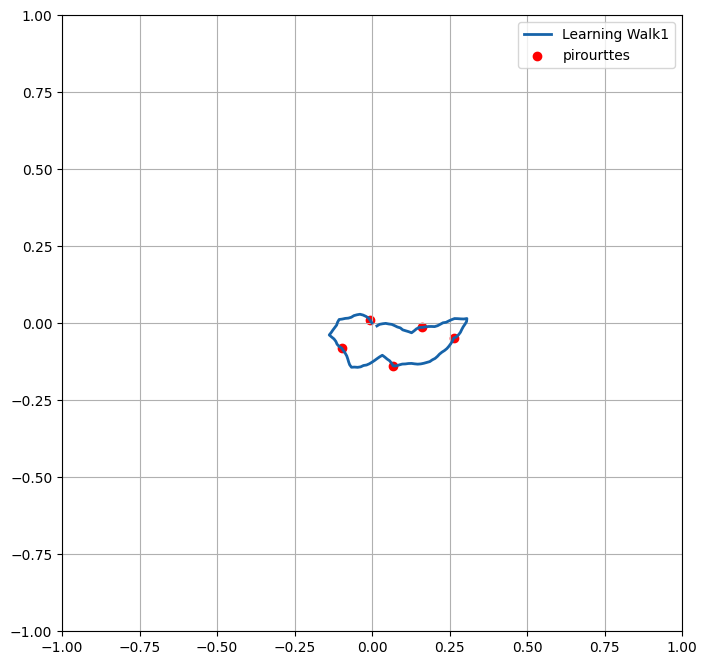

Supplement: Supplementary file 1 [file biomimetics-10-00736-s001.zip › biomimetics-3867713-supplementary/Supplement_Asymmetric olfactory field/FigureS5.png]

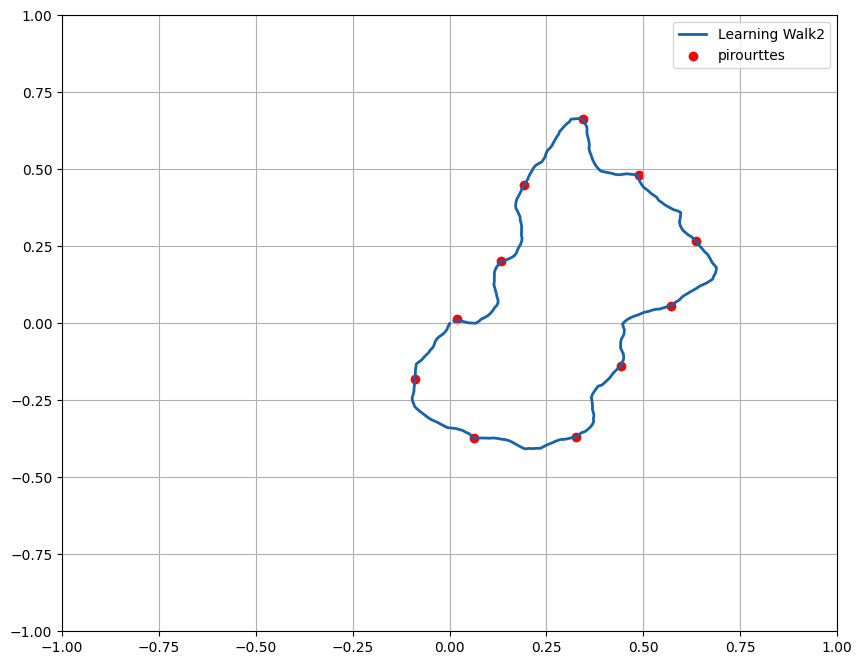

Supplement: Supplementary file 1 [file biomimetics-10-00736-s001.zip › biomimetics-3867713-supplementary/Supplement_Asymmetric olfactory field/FigureS6.png]

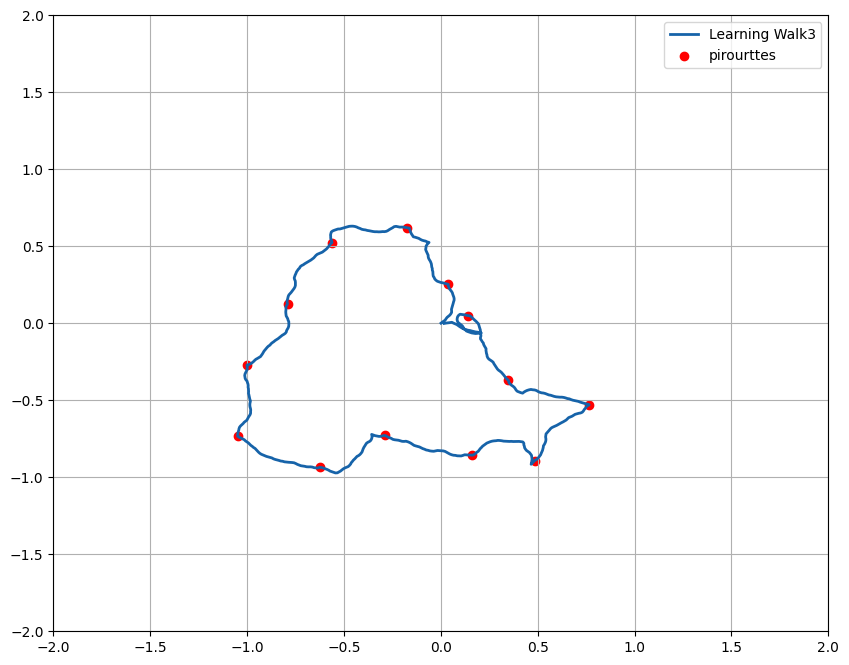

Supplement: Supplementary file 1 [file biomimetics-10-00736-s001.zip › biomimetics-3867713-supplementary/Supplement_Asymmetric olfactory field/FigureS7.png]

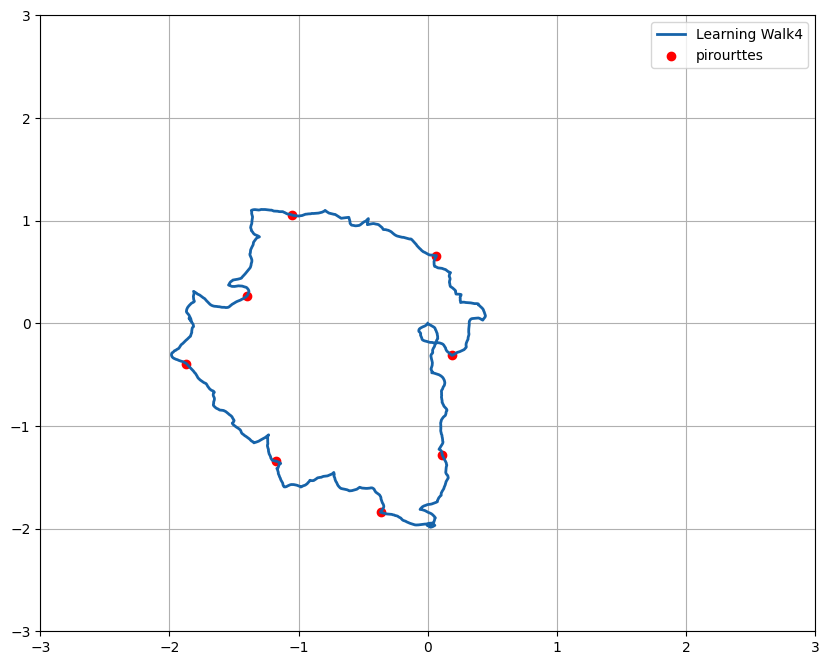

Supplement: Supplementary file 1 [file biomimetics-10-00736-s001.zip › biomimetics-3867713-supplementary/Supplement_Asymmetric olfactory field/FigureS8.png]

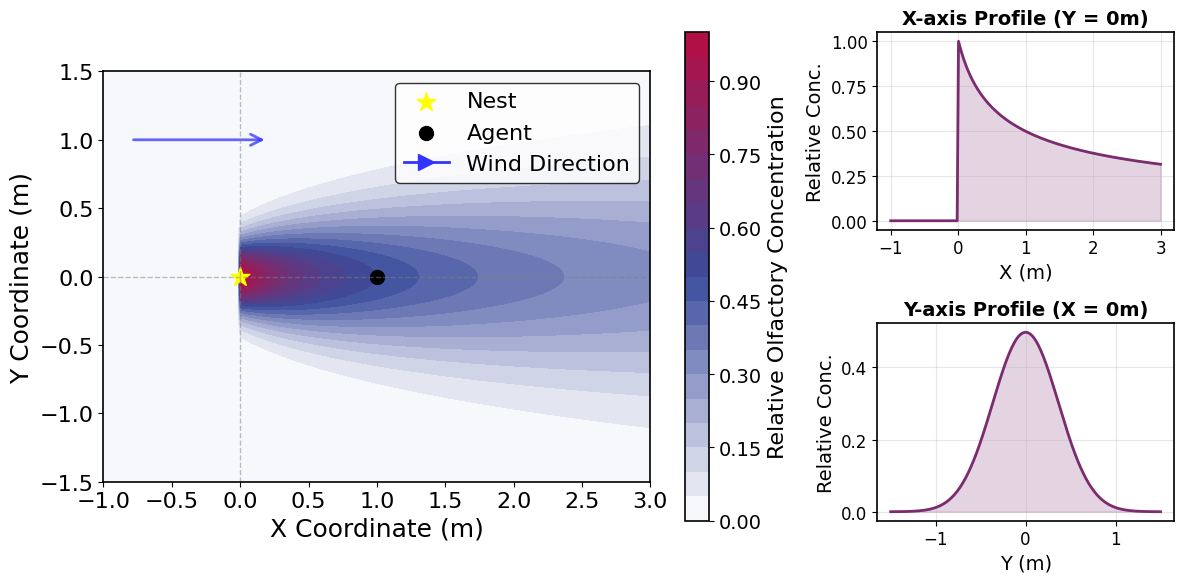

Supplement: Supplementary file 1 [file biomimetics-10-00736-s001.zip › biomimetics-3867713-supplementary/Supplement_Asymmetric olfactory field/FigureS9.png]

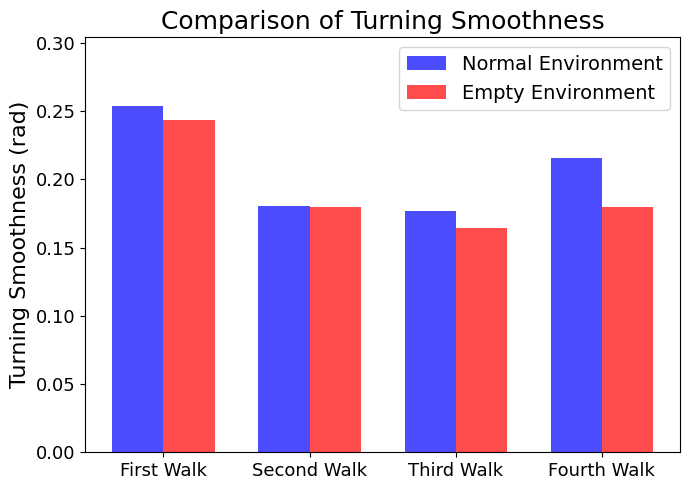

Supplement: Supplementary file 1 [file biomimetics-10-00736-s001.zip › biomimetics-3867713-supplementary/Supplement_Empty_World/FigureS10.png]

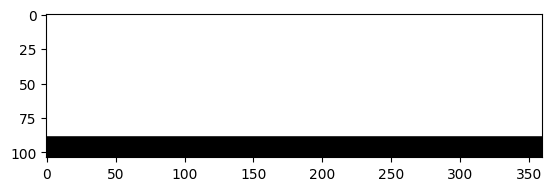

Supplement: Supplementary file 1 [file biomimetics-10-00736-s001.zip › biomimetics-3867713-supplementary/Supplement_Empty_World/FigureS11.png]

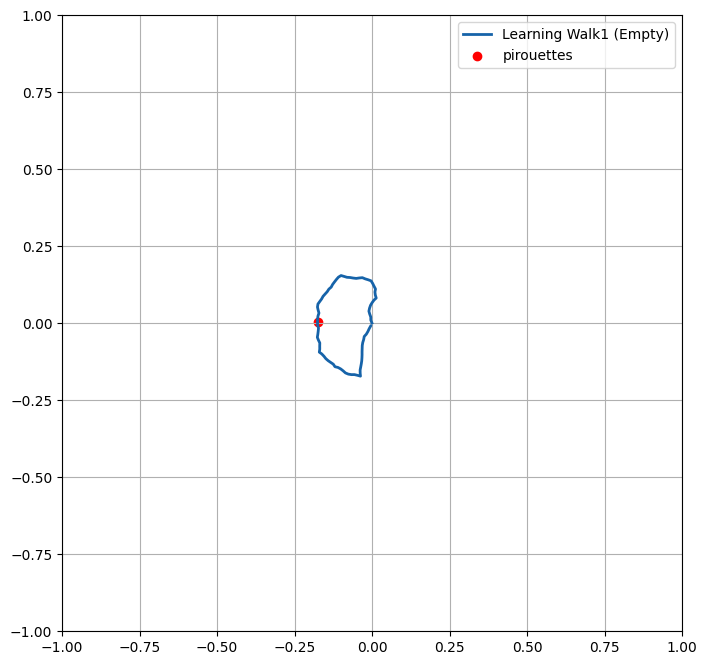

Supplement: Supplementary file 1 [file biomimetics-10-00736-s001.zip › biomimetics-3867713-supplementary/Supplement_Empty_World/FigureS12.png]

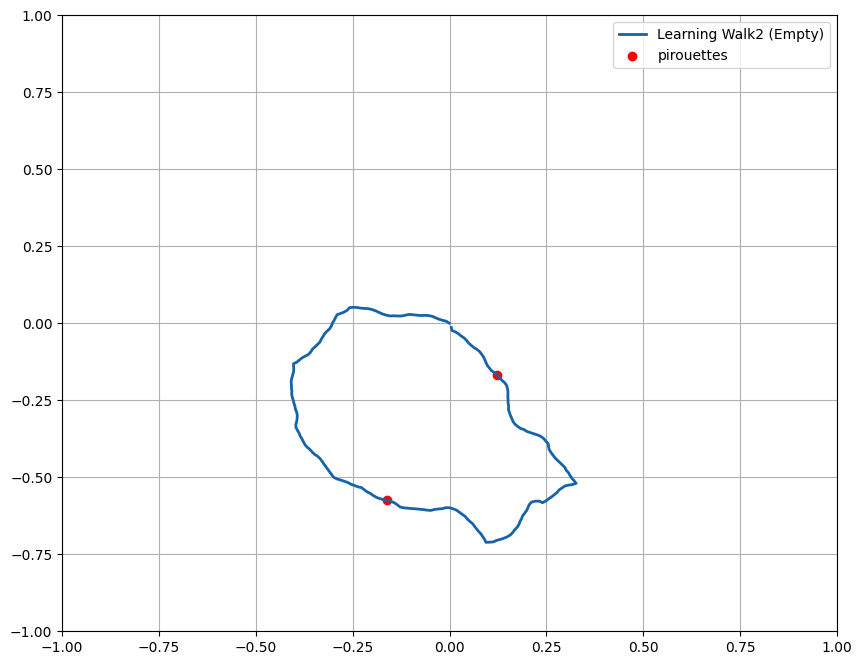

Supplement: Supplementary file 1 [file biomimetics-10-00736-s001.zip › biomimetics-3867713-supplementary/Supplement_Empty_World/FigureS13.png]

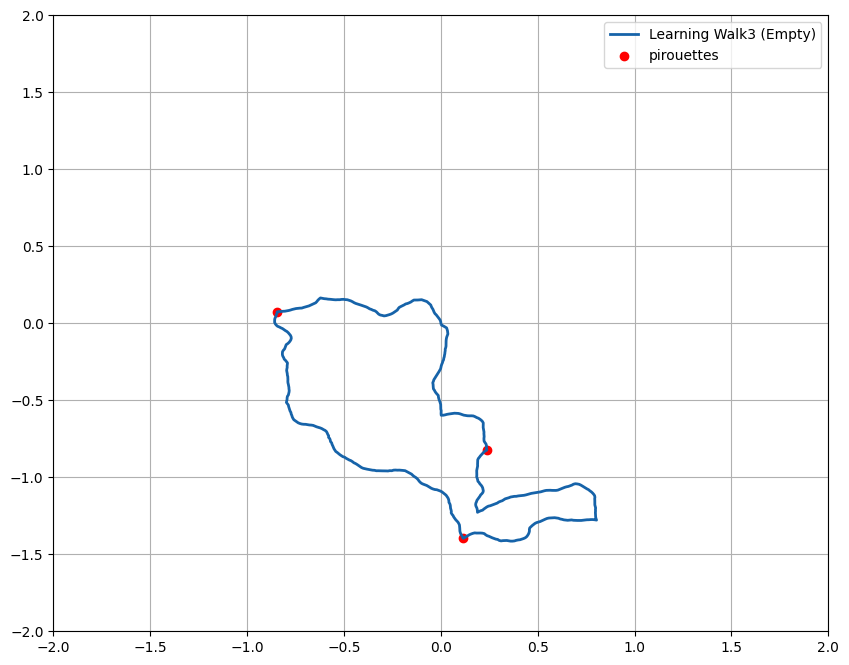

Supplement: Supplementary file 1 [file biomimetics-10-00736-s001.zip › biomimetics-3867713-supplementary/Supplement_Empty_World/FigureS14.png]

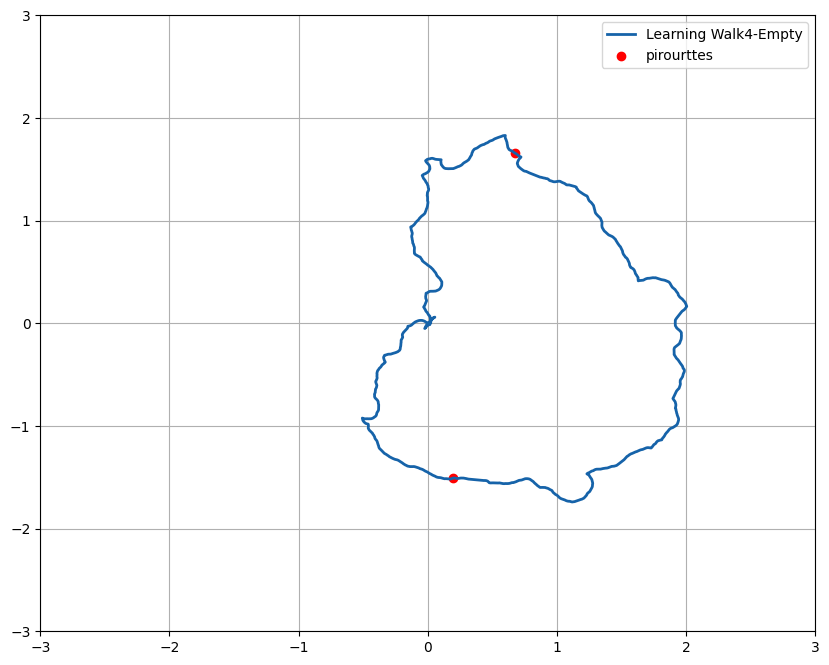

Supplement: Supplementary file 1 [file biomimetics-10-00736-s001.zip › biomimetics-3867713-supplementary/Supplement_Empty_World/FigureS15.png]

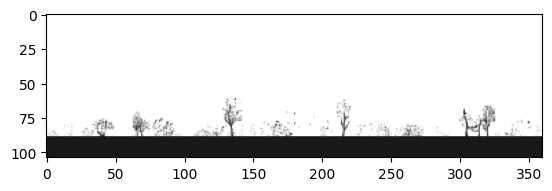

Supplement: Supplementary file 1 [file biomimetics-10-00736-s001.zip › biomimetics-3867713-supplementary/Supplement_Empty_World/FigureS16.png]

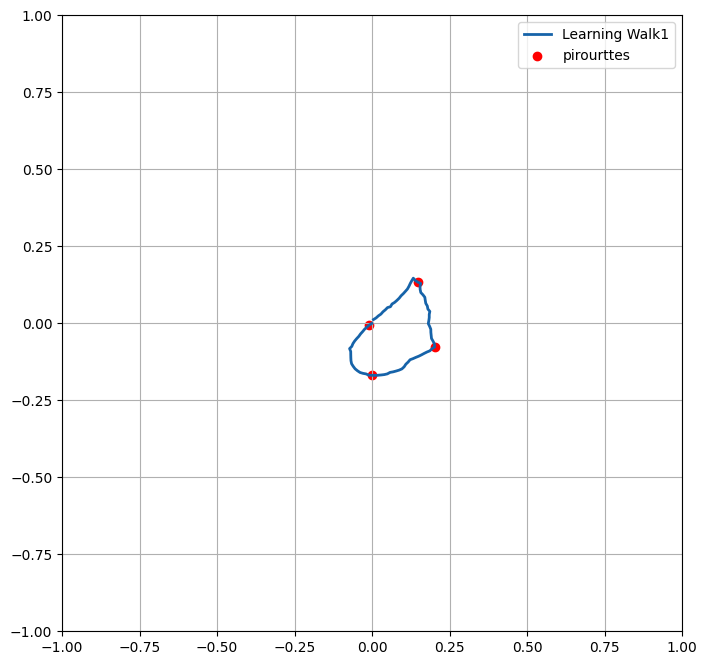

Supplement: Supplementary file 1 [file biomimetics-10-00736-s001.zip › biomimetics-3867713-supplementary/Supplement_Empty_World/FigureS17.png]

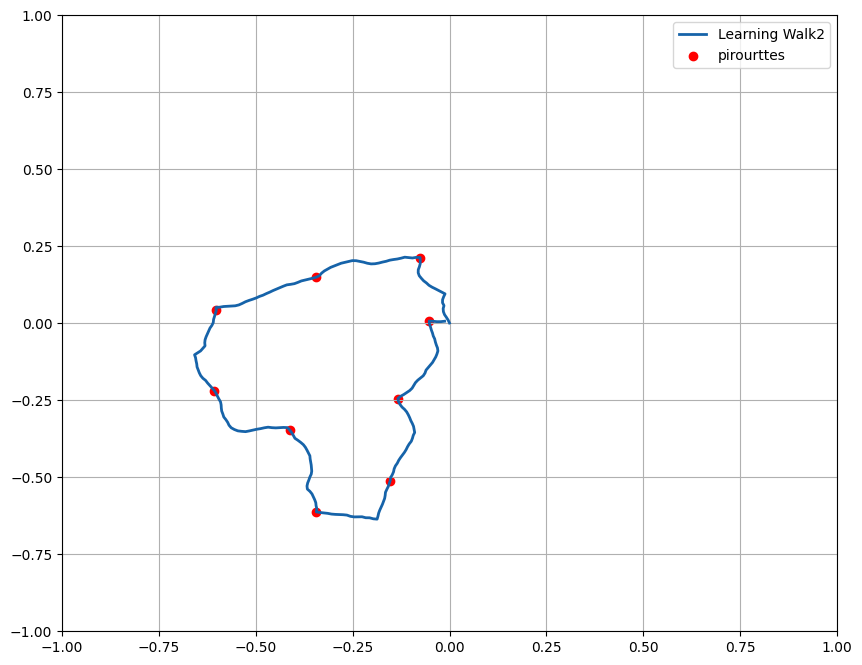

Supplement: Supplementary file 1 [file biomimetics-10-00736-s001.zip › biomimetics-3867713-supplementary/Supplement_Empty_World/FigureS18.png]

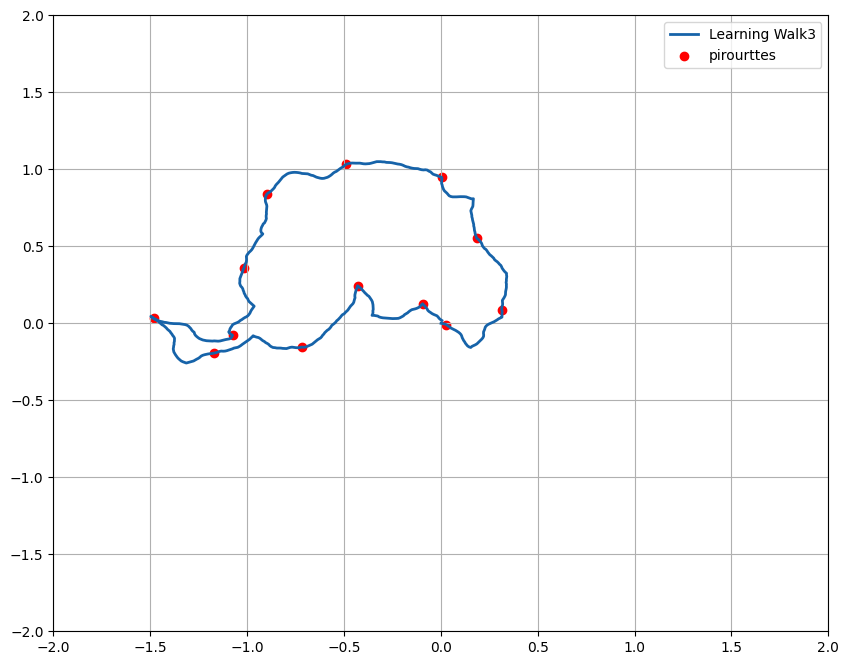

Supplement: Supplementary file 1 [file biomimetics-10-00736-s001.zip › biomimetics-3867713-supplementary/Supplement_Empty_World/FigureS19.png]

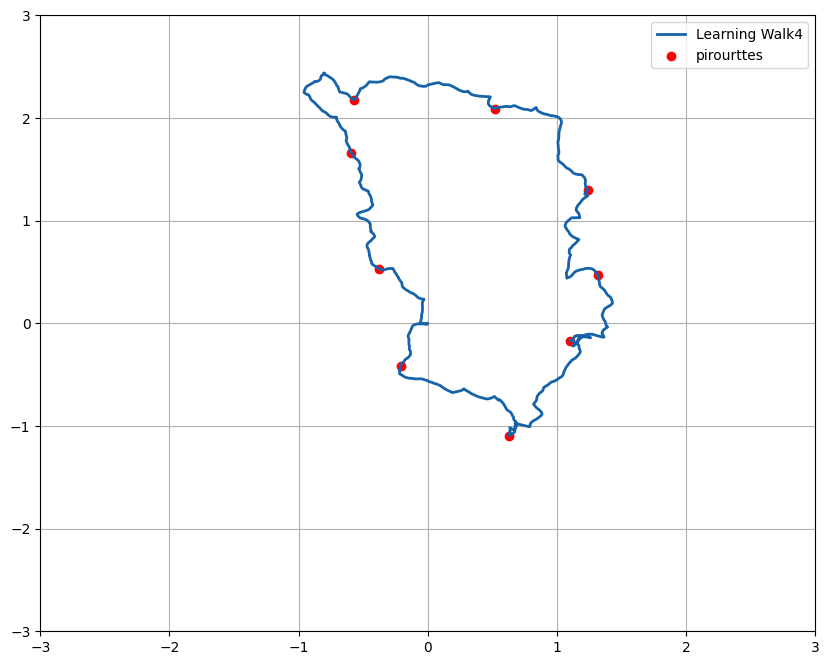

Supplement: Supplementary file 1 [file biomimetics-10-00736-s001.zip › biomimetics-3867713-supplementary/Supplement_Empty_World/FigureS20.png]

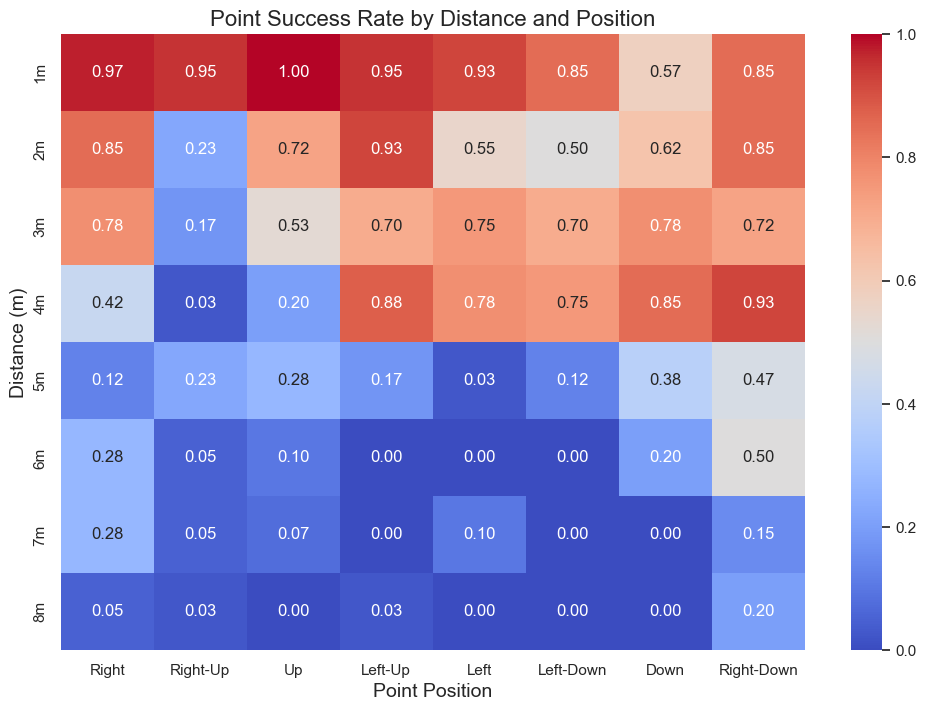

Supplement: Supplementary file 1 [file biomimetics-10-00736-s001.zip › biomimetics-3867713-supplementary/Supplement_Figure4/FigureS21.png]

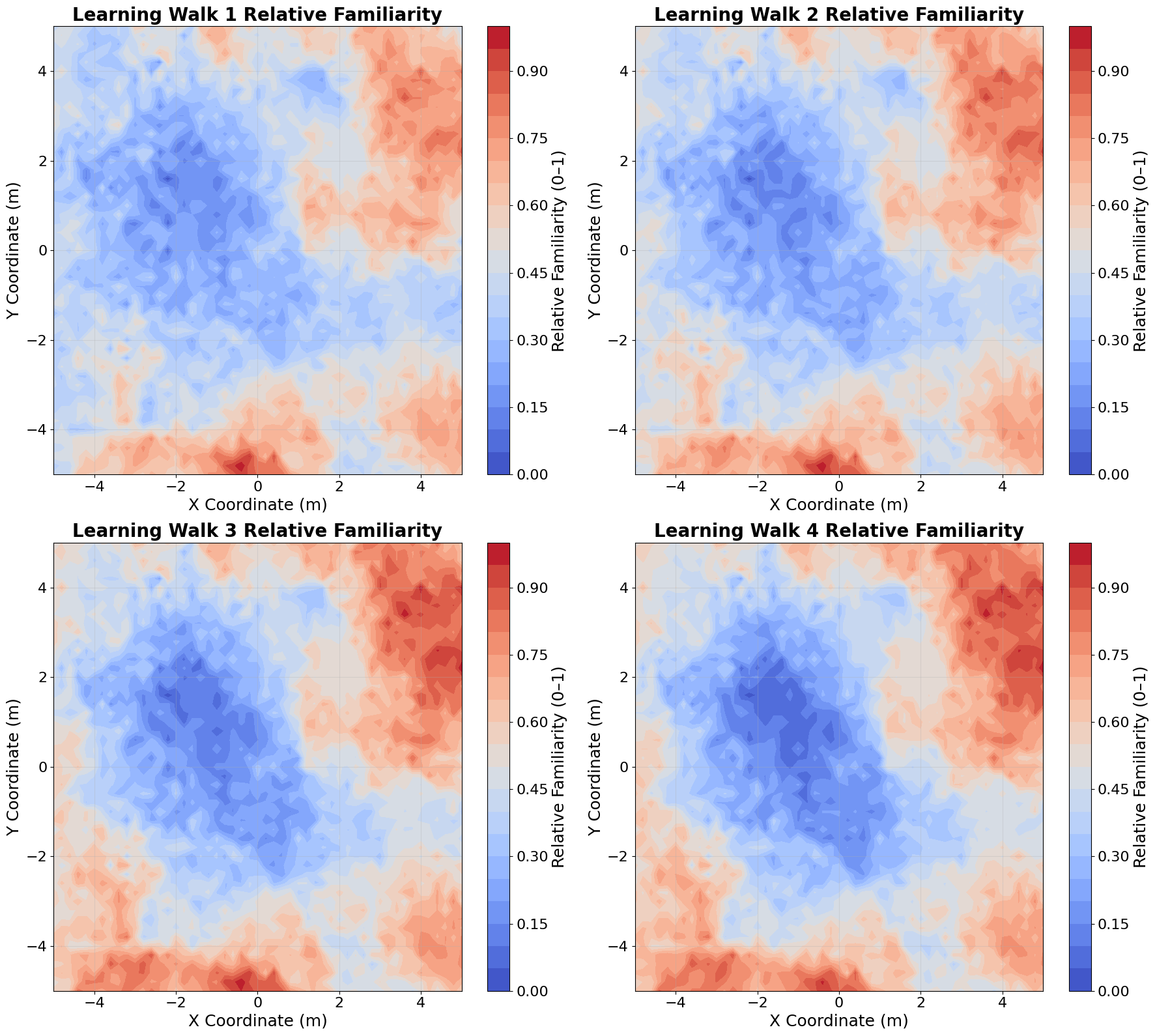

Supplement: Supplementary file 1 [file biomimetics-10-00736-s001.zip › biomimetics-3867713-supplementary/Supplement_Figure4/FigureS22.png]

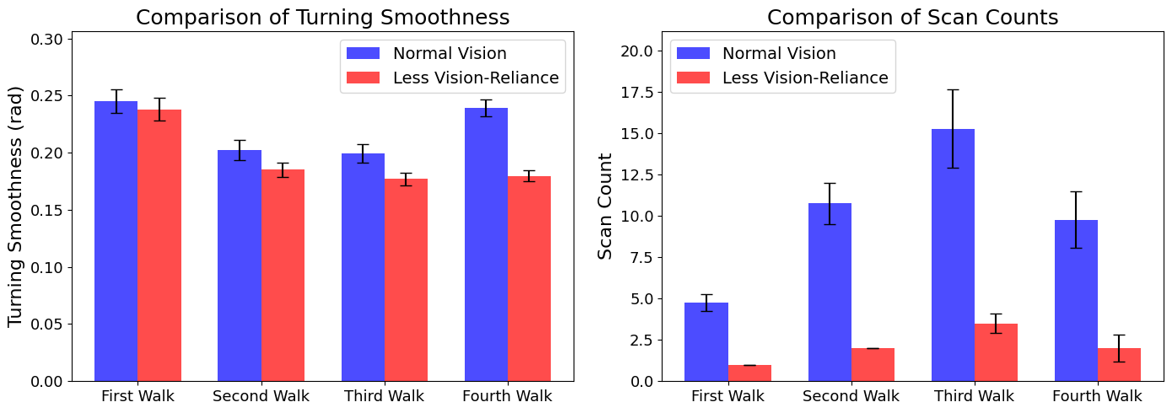

Supplement: Supplementary file 1 [file biomimetics-10-00736-s001.zip › biomimetics-3867713-supplementary/Supplement_Tortuosity/FigureS23.png]

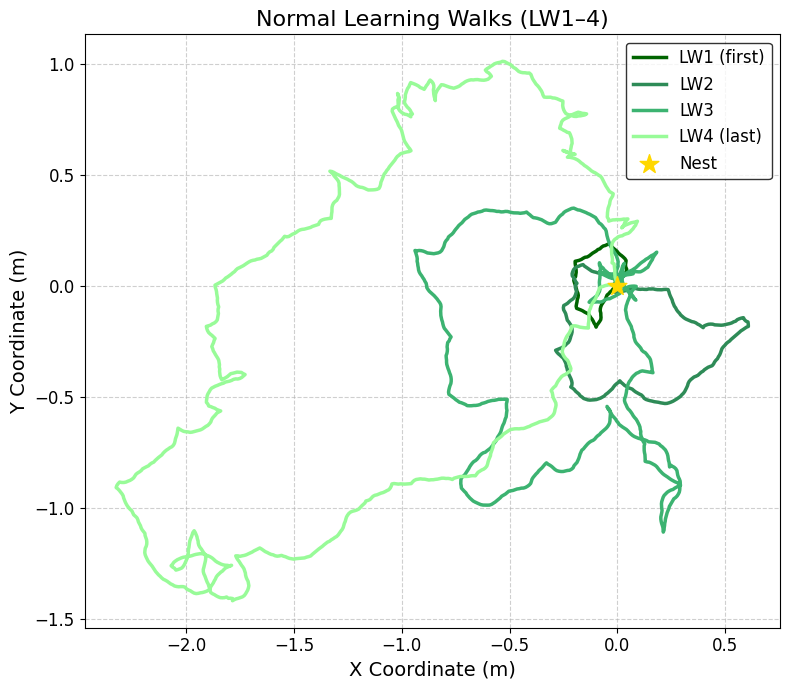

Supplement: Supplementary file 1 [file biomimetics-10-00736-s001.zip › biomimetics-3867713-supplementary/Supplement_Tortuosity/FigureS24.png]

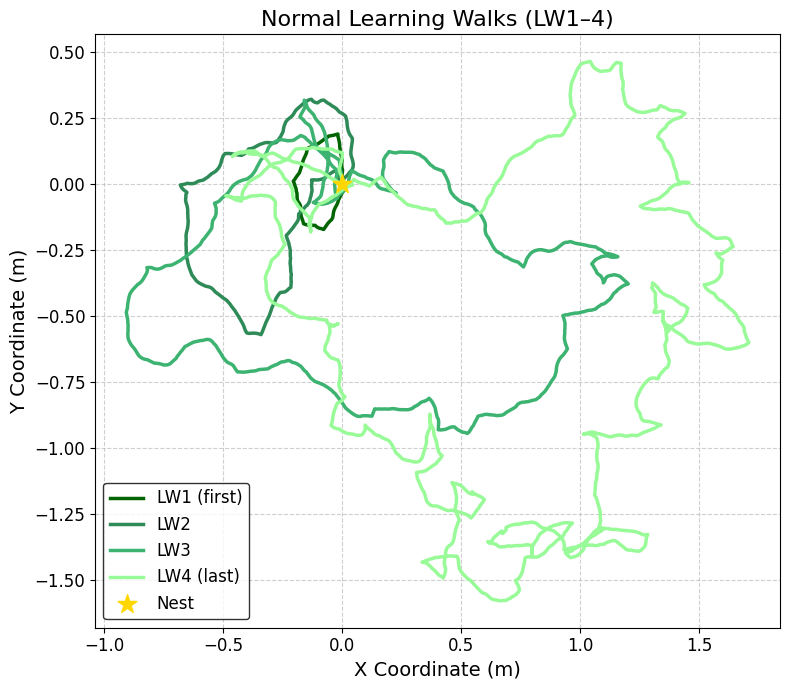

Supplement: Supplementary file 1 [file biomimetics-10-00736-s001.zip › biomimetics-3867713-supplementary/Supplement_Tortuosity/FigureS25.png]

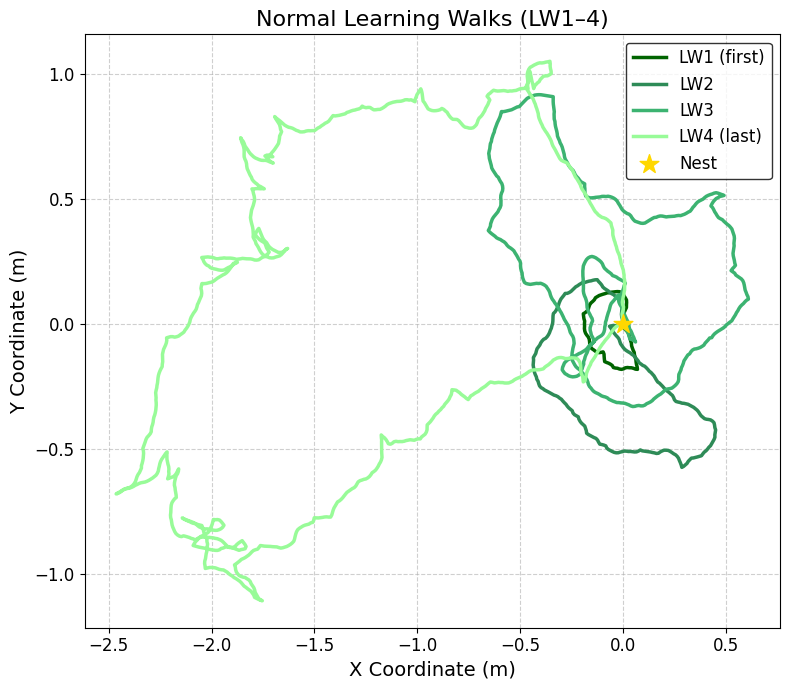

Supplement: Supplementary file 1 [file biomimetics-10-00736-s001.zip › biomimetics-3867713-supplementary/Supplement_Tortuosity/FigureS26.png]

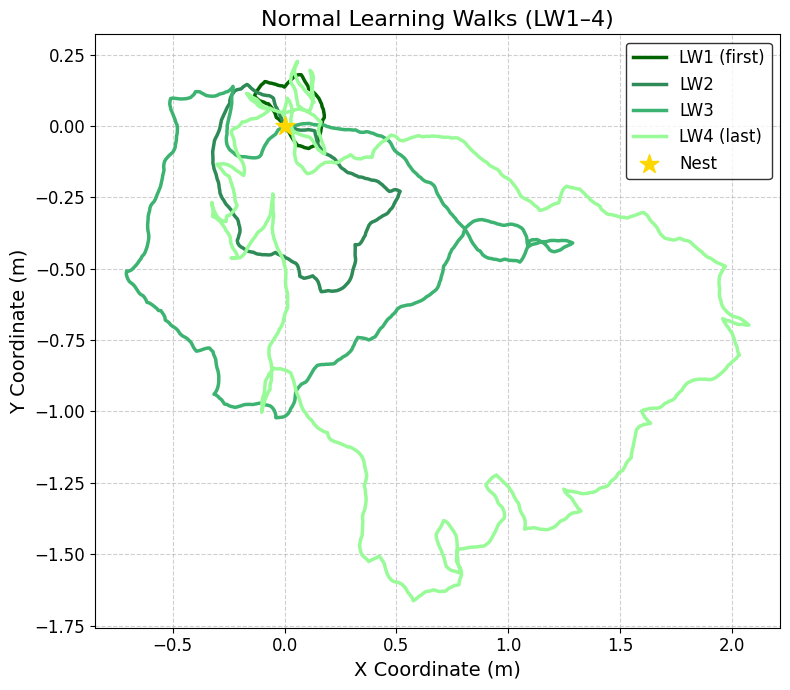

Supplement: Supplementary file 1 [file biomimetics-10-00736-s001.zip › biomimetics-3867713-supplementary/Supplement_Tortuosity/FigureS27.png]

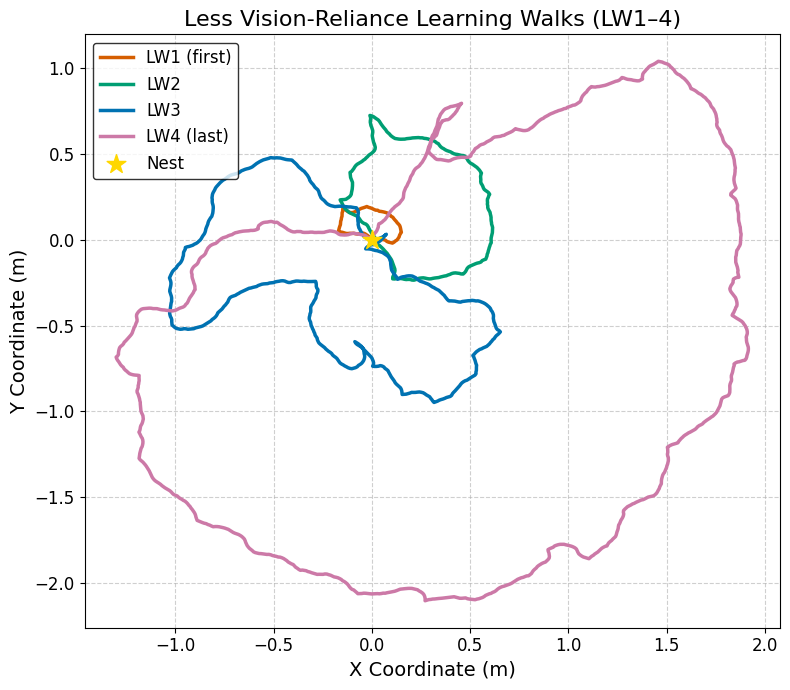

Supplement: Supplementary file 1 [file biomimetics-10-00736-s001.zip › biomimetics-3867713-supplementary/Supplement_Tortuosity/FigureS28.png]

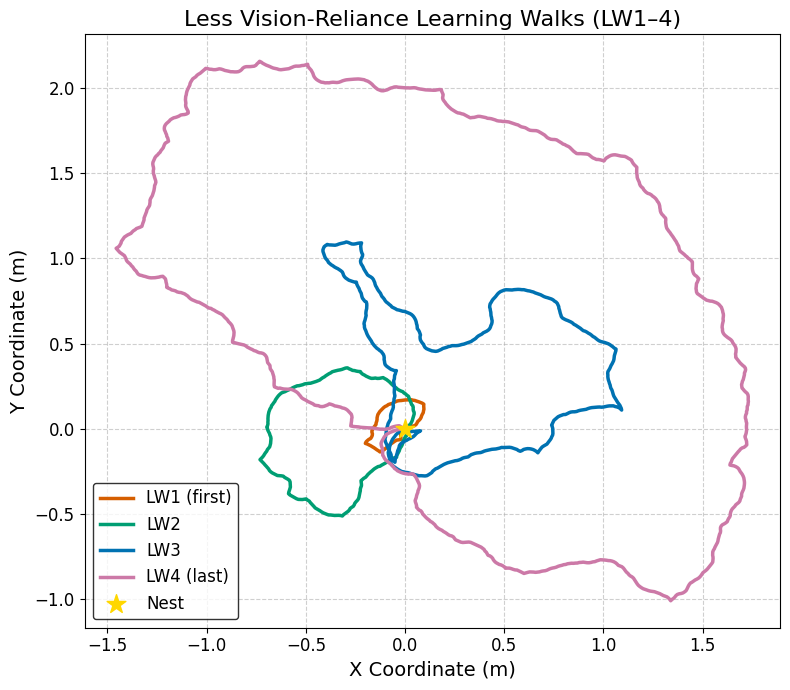

Supplement: Supplementary file 1 [file biomimetics-10-00736-s001.zip › biomimetics-3867713-supplementary/Supplement_Tortuosity/FigureS29.png]

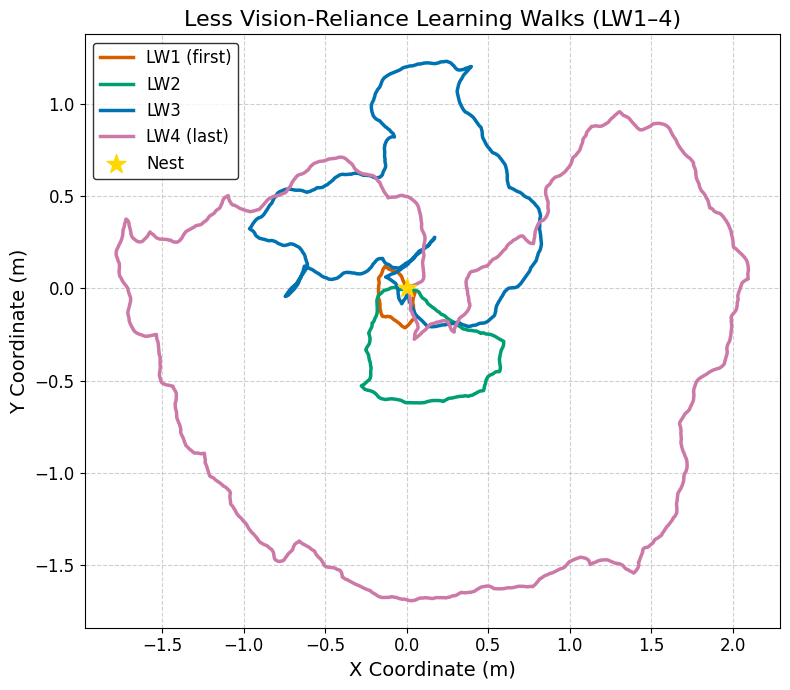

Supplement: Supplementary file 1 [file biomimetics-10-00736-s001.zip › biomimetics-3867713-supplementary/Supplement_Tortuosity/FigureS30.png]

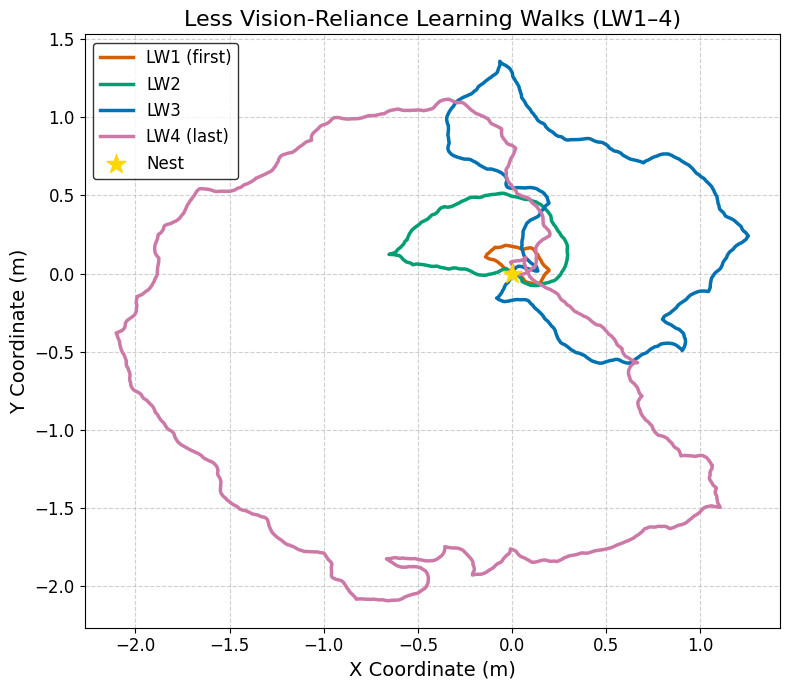

Supplement: Supplementary file 1 [file biomimetics-10-00736-s001.zip › biomimetics-3867713-supplementary/Supplement_Tortuosity/FigureS31.png]
